# Constitutive Innate Immunity and Systemic Responses to Infection of the American Alligator (*Alligator mississippiensis*)

**DOI:** 10.3390/ani14060965

**Published:** 2024-03-20

**Authors:** Mark Merchant, Matthew Hebert, Anna C. Salvador, Jennifer Berken, Thomas Boverie, Mary E. White

**Affiliations:** 1 Department of Chemistry, McNeese State University, Lake Charles, LA 70605, USAtgboverie@camtel.net (T.B.); 2 Department of Nutrition and Food Science, McNeese State University, Lake Charles, LA 70605, USA; 3 Department of Mathematical Sciences, McNeese State University, Lake Charles, LA 70605, USA; jberken@mcneese.edu; 4 Department of Biological Sciences, Southeastern Louisiana University, Hammond, LA 70402, USA; mary.white@selu.edu

**Keywords:** alligator, infection, inflammation, crocodylian, crocodilian

## Abstract

**Simple Summary:**

Alligators (*Alligator mississippiensis*) exhibit unusual immune responses relative to endothermic organisms. For example, most of the highest-expressed liver genes in uninfected mice are related to metabolism. In contrast, the most abundantly expressed hepatic genes expressed in alligators are immune system-oriented. The constraints of endothermy demand a high expression of metabolic genes to maintain temperature and elevated metabolism to support endothermy, while ectothermic alligators reallocate those resources to maintain a highly primed immune system. Injection of alligators with 200× the LD_50_ of bacterial lipopolysaccharide in mice resulted in no observable biological effects that are observed in endotherms. In addition, treatment with live bacteria caused a decrease in metabolic rate, which again, is the opposite of that exhibited by endotherms. The decrease in oxygen consumption was not accompanied by changes in heart rate or respiration, and thus could not be attributed to bradycardia or bradypnea. Injection of alligators with bacteria caused a complete elimination of digestive tract contents within a few hours. We interpret these activities as temporary minimization of other biological systemic activities to redirect and devote energy to immune function. The reallocation of resources within an organism to fight infection without increases in metabolic rate has not been described in other animals.

**Abstract:**

Uninfected alligators (*Alligator mississippiensis*) exhibited high constitutive levels of hepatic gene expression related to immune function, whereas the highest-expressed hepatic genes of uninfected mice were related to metabolism. Intraperitoneal challenge of mice with bacterial lipopolysaccharide results in dramatic inflammatory effects including peritoneal ascites, febrile response, dramatic alterations in electrophoretic serum profile, and mortality. In contrast, coelomic injection of alligators with 200× the murine LD_50_ of intraperitoneal bacterial lipopolysaccharide resulted in no changes in serum protein profiles, behavioral effects, mortality, and no coelomic ascites. However, injection of juvenile alligators with live bacteria resulted in a titer-dependent decrease in metabolic rate, as measured by oxygen consumption. These results are the opposite of those observed for mammalian and avian species. The decreased oxygen consumption was not accompanied by changes in heart or respiration rate, indicating that this phenomenon was not due to bradycardia or bradypnea. Interestingly, challenge of alligators with bacteria resulted in the complete expulsion of digestive tract contents within four hours. We interpret these activities as temporary minimization of other biological systemic activities to redirect and devote energy to immune function. The reallocation of resources within an organism to fight infection without increases in metabolic rate has not been described in other animals.

## 1. Introduction

Immune performance is a physiological and biochemical trait that is vital to fitness and survival [1]. The energy required to maintain effective immunity, respond to infection, and effectively clear potential pathogens is critical for the biological success of an individual and/or species [2]. Immune fitness is temperature-sensitive in both endotherms [3] and ectotherms [4]. While endotherms normally only experience small diurnal [5] and seasonal variations [6] in body temperature, ectotherms can exhibit large internal temperature ranges and have much more complex thermal ecology [7]. Selection for thermal peak performance of biological function (such as immune function) is typically strong for homothermic species, but usually is broad for heterothermic species [8].

Endotherms exhibit well-developed immune systems that rely on both innate immune mechanisms and advanced adaptive mechanisms [9]. Innate immune mechanisms act as a first line of defense against infection and are typically rapid but nonspecific in nature [10]. In contrast, adaptive immunity is more specific, requires previous exposure, and takes several days to develop a full response [11]. Most ectotherms rely heavily on innate immunity and have less complex adaptive immunity than endotherms [9]. In addition, ectotherms can have limited resources available to provide energy for immunity. It is thought that the energetic constraints of ectothermy limit the development and maintenance of energy-expensive adaptive immune mechanisms [8]. Interestingly, species with broader thermal ranges exhibit higher peak performances of innate immune parameters [8]. This could be a compensatory mechanism for the lack of complex adaptive immunity.

American alligators (*Alligator mississippiensis*) have potent and complex innate immune systems that include phospholipase A_2_ [12], dipeptidyl peptidase [13], and serum complement activities [14]. Furthermore, alligators utilize iron withholding [15] and behavioral febrile responses [16] to combat infection. However, these animals have less complex immune systems than endothermic vertebrates [17]. For instance, alligators have less-developed T-cells and B-cells [18] that respond to a variety of mitogenic stimulants [19]. In addition, alligators express immunoglobulin Y (IgY) [20], an early class of antibodies produced by amphibians, reptiles, and birds that is an ancestral ortholog to IgG produced in higher vertebrates [21]. Alligators also do not have lymph nodes, germinal centers, or Peyer’s patches, as these structures are only found in endothermic vertebrates [18].

The objectives of this study were to characterize the systemic immune response of American alligators by artificially inducing an infection and measuring direct and indirect correlates of immune function. Direct measurements included immune proteins in blood serum and analysis of the liver transcriptome, and indirect measurements included oxygen consumption, heart rate, respiratory rate, and fecal output. We also characterized constitutive hepatic gene expression in both mice and alligators to determine the basal levels and functions of the highest-expressed genes.

## 2. Materials and Methods

**Biochemicals**—Lyophilized *Klebsiella oxytoca*, *Staphylococcus aureus, Escherichia coli* and *Salmonella typhi* bacteria, and lipopolysaccharide (LPS) derived from *E. coli* were purchased from Remel, Inc. (Lake Charles, LA, USA), reconstituted, and used immediately upon arrival. Bactoagar was purchased from Millipore Sigma (St. Louis, MO, USA).

**Treatment of animals**—For the experiments in which electrophoretic analysis of serum is described, 8 juvenile alligators (72–78 cm total length) were captured by hand from a boat at McFaddin National Wildlife Refuge (12 miles east of Sabine Pass, TX, USA) and transported to our laboratory at McNeese State University in Lake Charles, Louisiana. Animals were maintained in a polypropylene tank (7000 cm^2^ surface area) with 50% water surface area of depth 0–8 cm (four animals per tank). The water was heated to 30 °C with submersible aquarium heaters. The animals were fed dried pelletized commercial alligator food (Burris Mill and Feed, Franklinton, LA, USA) ad libitum daily, and tanks were cleaned daily.

A total of 24 alligators were used for oxygen consumption experiments (72–78 cm total length); they were captured by hand at McFaddin National Wildlife Refuge and transported to our laboratory at McNeese State University in Lake Charles, LA, USA. The animals were maintained and fed in the same manner as those described above.

Mice were housed in cages in the vivarium of the Biology Building at Southeastern Louisiana University and fed ad libitum.

**Serum electrophoresis**—Whole blood was collected from alligators via the spinal vein [22] using 2.5 cm, 22 ga needles and 3 mL syringes. The blood was allowed to clot at room temperature for approximately one hour, and then centrifuged at 5000× *g* (ambient temperature) to collect the serum. The serum (10 μL) was loaded onto an application plate of an automated SPIFE™ electrophoresis instrument (Helena Laboratories, Beaumont, TX, USA). The serum was autoloaded onto the gel and the proteins were resolved after electrophoresis for 15 min. The proteins were manually stained in approximately 100 mL of 5% acetic acid/30% methanol for 10 min and then destained in 10% acetic acid and 50% methanol (*v*/*v*) for 10 min. The gel was dried on the SPIFE instrument and scanned using a Quickscan 2000™ digital densitometer (Helena Laboratories, Beaumont, TX, USA). The relative amounts of α globulins, β globulins, γ globulins, and albumin were determined by the Quickscan software.

Systemic effects of endotoxin were measured by the injection of high doses of LPS (50, 100, and 500 mg/kg) derived from *E. coli* in the coelomic cavity of juvenile alligators. Attempts to collect ascites fluid were conducted using 2.5 cm 18 ga needles and 3 mL syringes 6, 12, 24, 48, and 72 h after injection of LPS. Whole blood was collected via the spinal vein, processed, and agarose gel electrophoresis was conducted as described above.

**Transcriptome**—Untreated alligators (*Alligator mississippiensis*) and mice (*Mus musculus*) were sacrificed by cervical dislocation, pithed, and their livers perfused with ice-cold isotonic saline via the hepatic portal vein. The livers were removed and approximately 50 mg was homogenized in 1.5 mL of ice-cold RNA*later*™ (Thermofisher Scientific, Waltham, MA, USA) with 20 strokes of a Dounce homogenizer. The homogenized samples were centrifuged at 16,000× *g* to remove insoluble material and the supernatant was transferred to 2 mL cryovials and stored at −80 °C until ready for use in transcriptome studies. Total hepatic RNA was isolated using a Qiagen RNeasy Plus Mini Kit per the manufacturer’s instructions (Qiagen, Austin, TX, USA). RNA quantity and integrity were measured using the Qubit 4 Fluorometer from Thermo-Fisher Invitrogen (Waltham, MA, USA). Novogene USA (Sacramento, CA, USA) performed library preparation, eukaryotic RNA sequencing, and preliminary analyses.

Purified RNA was submitted to Novogene USA for library construction and sequencing. Total RNA was enriched for mRNA using oligo(dT) beads. The mRNA was randomly fragmented, followed by first strand cDNA synthesis using random hexamers and reverse transcriptase. After first-strand synthesis, synthesis buffer (Illumina) was used to generate the second strand by nick-translation using *E. coli* polymerase I, dNTPs, and RNase H1. The final cDNA library underwent purification, terminal repair, A-tailing, ligation of sequencing adapters, size selection, and PCR enrichment.

Libraries were sequenced using Illumina sequencing. Raw reads were filtered to remove low quality sequences containing adaptors or with more than 10% uncertain nucleotides. Over 95% of the original reads were clean reads.

Sequences were mapped to reference genomes using the HISAT2 algorithm. Gene expression was quantified using the fragments per kilobase of transcript sequence per million base pairs sequenced (FPKM) method.

**Oxygen consumption**—Oxygen chambers were constructed using 60 cm lengths of 10 cm diameter PVC pipe. The end of each chamber was fitted with a PVC cap that was glued to seal one end while the other end contained a removeable cap. Two holes of 3.5 mm were drilled approximately 8 cm from each end of the chamber, and a threaded bronze barbed hose nozzle was screwed into each hole using teflon tape to ensure an airtight seal. The tubes of the ADInstruments Powerlab 4/26 (Colorado Springs, CO, USA) respiratory gas analyzer were connected to the hose nozzle. The chambers were wrapped with electric heated blankets to maintain a constant internal temperature of 30 °C. These chambers provided a dark, quiet, constant-temperature environment in which we measured metabolic parameters. Oxygen consumption was measured in each alligator on three consecutive days to obtain baseline metabolism values for each animal.

To measure the effects of infection on oxygen consumption, four alligators were left untreated, four were injected in the coelomic cavity with 250 μL of sterile saline, and four each were injected with 250 μL of saline that each contained five-fold serial dilutions (1 × 10^7^, 2 × 10^6^, 4 × 10^5^, or 8 × 10^4^ CFU) of equal parts *Klebsiella oxytoca*, *Staphylococcus aureus*, *Escherichia coli*, and *Salmonella typhi* bacteria. Immediately after injection, an alligator was placed in the chamber and the cap was placed on the end. Air entered the chamber through the hose nozzle closest to the tail at a flow rate of 200 mL/min, flowed past the animal, and out the nozzle closest to the head of the animal. Once outside the chamber, the air passed through a cartridge containing 25 g of anhydrous CaCl_2_ drying agent to remove moisture [23]. The animal was allowed to acclimate for approximately 15 min to the environment until its oxygen consumption was stable and minimal. After this time, the oxygen levels were recorded and the alligator was removed from the chamber and returned to its tank. The data from this trial were used as time 0, and this procedure was repeated for each animal at 12, 24, 48, 72, and 96 h after injection. The metabolic rate was calculated considering the oxygen consumption at each time point and the mass of each animal. Because the animals were at rest and anaerobic metabolism was not a factor, the oxygen consumption (mL O_2_/min) was converted to J/min by multiplying by 20.1 J/mL O_2_ [24,25] and 60 min/h and dividing by the mass of the animals in kg, providing the metabolic rate expressed as J/h/kg body mass. The metabolic rate of each alligator at the different time points were compared by that obtained for that animal at time 0, and thus the time 0 value was the internal control for each individual animal.

**Heart rate**—Heart rate was measured in alligators using a noninvasive heart rate monitor accessory with the ADInstruments Powerlab. The heart rate sensor was secured around the midsection of each animal using an expandable elastic belt with Velcro. The sensor was positioned between the two legs of each alligator, as close to the heart as possible, for maximum sensitivity. Each animal was placed in the PVC chamber and heart rate was measured until minimal and stable for at least five min. The baseline heart rate was recorded for each animal.

**Respiratory rate**—The respiratory rate was measured using a respiratory rate belt transducer attachment for the ADInstruments Powerlab 4/26 analyzer. The belt was secured to the animal around the thoracic cavity using the elastic belt and secured with Velcro, and chest expansion were measured until breathing became stable, which usually required 10–15 min, at which time the respiratory rate was recorded.

**Fecal output**—Twelve alligators were fed ad libitum, isolated after feeding to individual tanks of 2.5 cm water depth, and the daily fecal output of each animal was measured on three consecutive days to obtain a baseline value for each animal. After 6 h, the water was removed and filtered through 0.45 μM filter paper in a 10 cm Buchner funnel fitted into a side-arm Erlenmeyer suction flask. The filter papers were dried in an oven at 50 °C and the mass of the fecal material for each animal was determined by subtraction of the predetermined masses of the filter papers. Four alligators were injected with a cocktail containing 10^5^ CFU each of *Klebsiella oxytoca*, *Staphylococcus aureus*, *Escherichia coli,* and *Salmonella typhi* bacteria in 250 μL isotonic saline, sterile isotonic saline, or left untreated, and then placed in 86 cm × 38 cm individual polypropylene tubs in approximately 2.5 cm water depth. The fecal output of each animal was measured 6 h post-treatment as described above.

**Statistics and Controls**—Most of the results reported are the means ± standard deviations of four independent determinations. Results of serum electrophoresis for each alligator after infection were compared to the results obtained for the same animal before treatment, and the globulin/albumin are expressed as percent change. The oxygen consumption rates were also measured prior to treatment and subsequent results were compared to the preliminary baseline results of each animal. In the same manner, heart rate and respiratory rates were also measured prior to the start of the experiment to obtain baseline results for each animal, and the experimental results are expressed as the percent change ± standard deviations for each treatment group. IBM SPSS (Version 28.0.1) software was used for assumption checks and analysis of data relating to heart rate, respiratory rate, metabolic rate, and fecal output. In each case, a repeated measures Analysis of Variance (ANOVA) model was conducted with treatment group as a between-subjects factor. For comparing treatment levels, pairwise comparison tests were used with a Sidak adjustment for multiple comparisons. For comparisons of time points within a treatment, Bonferroni corrections were used. Because the highest dose of bacteria (10^7^ CFU) injected resulted in the death of several alligators after the 24 h time point, the analysis of the results for this experiment was conducted in two different ways. The first analysis removed the 10^7^ CFU treatment group so that the entire time course could be analyzed, and the second analysis removed the 48–96 h time points so that the 10^7^ CFU treatment group could be compared to the untreated control group.

## 3. Results

**Figure 1:**
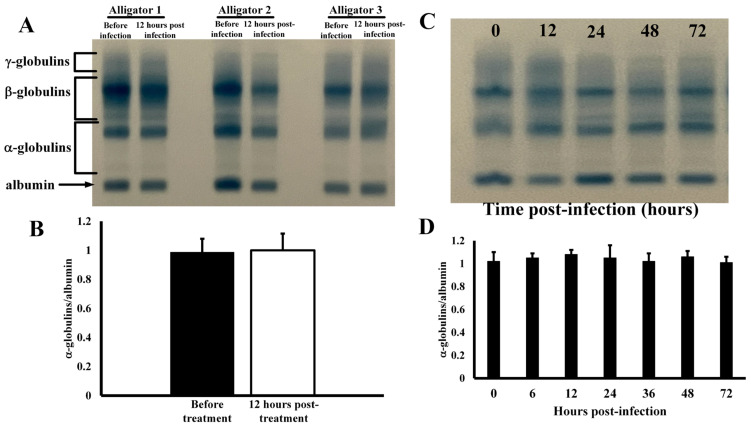
Electrophoretic profiles of serum from alligators. (**A**) Serum proteins profiles before and 12 h after coelomic injection of bacteria. (**B**) Globulin/albumin ratios derived from integration of densitometric scans of serum protein electrophoretic profiles. (**C**) Electrophoretic profiles of serum from an alligator after different time points after infection. (**D**) Globulin/albumin ratios (at different time points post-infection) derived from densitometric scans of serum protein electrophoretic profiles of alligators injected with bacteria.

**Effects of Infection on Serum Protein Profiles**—Co-injection of alligators in the coelomic cavity with four different types of bacteria resulted in no changes in serum protein electrophoretic profile (Figure 1). The serum profiles for several different animals exhibited no changes in any of the main serum protein groups (Figure 1A) before and after infection as densitometric analyses showed similar quantification, as percent of total serum protein, of the major protein groups (Figure 1B). The α-globulins constituted 1.7 ± 0.3% of all total serum proteins in untreated animals, and 1.7 ± 0.5% after infection (*p* = 0.92). Likewise, the β-globulins were 28.8 ± 6.4% of total serum protein before treatment and 23.0 ± 6.3% after infection (*p* = 0.88). The expression of γ-globulins was also not affected by injection with bacteria before (59.8 ± 9.7%) and after infection (61.1 ± 9.8%, *p* = 0.87). In addition, the globulin/albumin ratios of 0.174 ± 0.043 and 0.168 ± 0.043 were also statistically indistinguishable (*p* = 0.89) for these samples. The serum protein profiles remained constant for 72 h after infection (Figure 1D), as the globulin/albumin ratios did not change at any time point (*p* > 0.05).

**Constitutive hepatic gene expression**—Examination of alligator and murine constitutive hepatic transcriptome data revealed striking differences in expression patterns. After exclusion of highly expressed ribosomal and mitochondrial housekeeping genes and albumin, 55 of the 100 highest-expressed hepatic genes in uninfected mice coded for proteins that exhibit metabolic function, while only 11 of the most abundant transcripts resulted in the expression of proteins with immune function (Table 1). In contrast, only 19 of the highest constitutively expressed hepatic genes in uninfected alligators were related to metabolism, while 29 coded for proteins that exhibit immune function. In terms of total transcripts (7,262,841) from the top 100 expressed murine hepatic genes, 4,153,760 (57.2%) were genes related to metabolic activity and 796,288 (11.0%) were for immune function. Analysis of transcripts from the 100 highest-expressed alligator hepatic genes (total transcripts = 4,449,289) revealed that only 870,276 (19.6%) coded for genes related to metabolic processes and 1,494,348 (33.6%) had immune function (Table 1).

**Figure 2:**
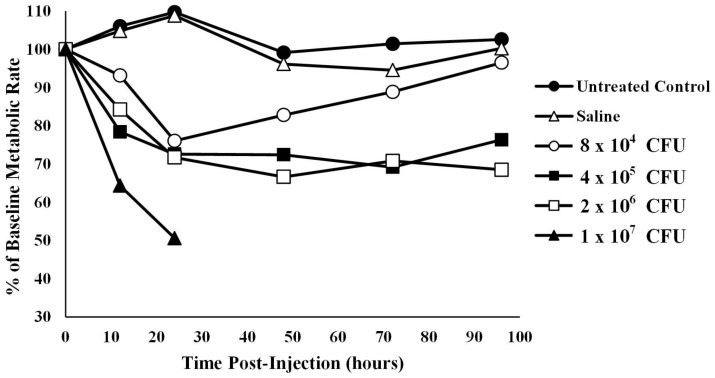
Metabolic rate, derived from oxygen consumption, of alligators infected with different titers of bacteria. The metabolic rate of untreated alligators or those treated with sterile saline were relatively stable. However, metabolic rate was decreased in a titer-dependent fashion in alligators infected with bacteria. The data set for 1 × 107 CFU is abbreviated as the alligators from this group succumbed to the infection after 24 h. The data are expressed as the means of four independent determinations.

**Oxygen consumption in infected alligators**—The average baseline metabolic rate for the 12 alligators used in this experiment was 14.20 ± 2.94 kJ/h/kg body mass at 30 °C. Coelomic injection of alligators with four different amounts of bacteria resulted in a titer- and time-dependent decrease in oxygen consumption (Figure 2). To account for missing data in the high bacterial treatment group (1 × 10^7^ CFU), two analyses were performed: one with the 1 × 10^7^ CFU group removed and one with hours 48, 72, and 96 removed. Mauchly’s test showed the assumption of sphericity had been violated (W = 0.391, *p* = 0.006) so a Greenhouse–Geisser correction was used. The assumption of normality was assessed using a Q-Q plot which showed a potential violation, so results should be interpreted with caution.

With the 1 × 10^7^ CFU treatment group removed, ANOVA test results showed that animals treated with sterile saline were not statistically different from untreated controls at any time point after time 0 (p12 h = 1.00, p24 h = 1.00, p48 h = 1.00, p72 h = 0.997, p96 h = 1.00). In addition, injection of alligators with the lowest bacterial titer (8 × 10^4^) resulted in metabolic rates that were not different from baseline levels at any time point (*p* > 0.05, Figure 2). However, injection of alligators with higher levels of bacteria (4 × 10^5^ CFU) caused a decrease in metabolic rate of 27.4% (*p* = 0.045) below baseline at 48 h, which stayed 30.8% (*p* = 0.07) and 23.7% (*p* = 0.04) below baseline at 72 and 96 h post-injection, respectively. Likewise, the metabolic rate for alligators injected with 2 × 10^6^ CFU exhibited metabolic rates 33.4% (0.013), 29.2% (*p* = 0.011), and 31.5% (*p* < 0.01) below baseline levels at 48, 72, and 96 h post-injection, respectively. Injection of alligators with the highest titer of bacteria (1 × 10^7^ CFU) resulted in a decrease of 35.7% (*p* < 0.01) and 49.4% (*p* < 0.01) at 12 and 24 h post-injection, respectively. Two of the alligators died between 24 and 36 h, and the third alligator in this group died between 36 and 48 h, which prevented further collection of data for this group.

One important aspect of these experiments was that alligators of very narrow size range were used, because as alligators grow larger their metabolic rates decrease [26]. Therefore, wild juvenile alligators of a narrow size range (72–78 cm total length) were used. It is important that the metabolic rates were standardized for each individual animal as oxygen consumption can vary between individual alligators of the same size by 20% [26]. In addition, although the metabolic rate of alligators is dependent on temperature, these animals do not exhibit seasonal metabolic rhythms [27].

**Figure 3:**
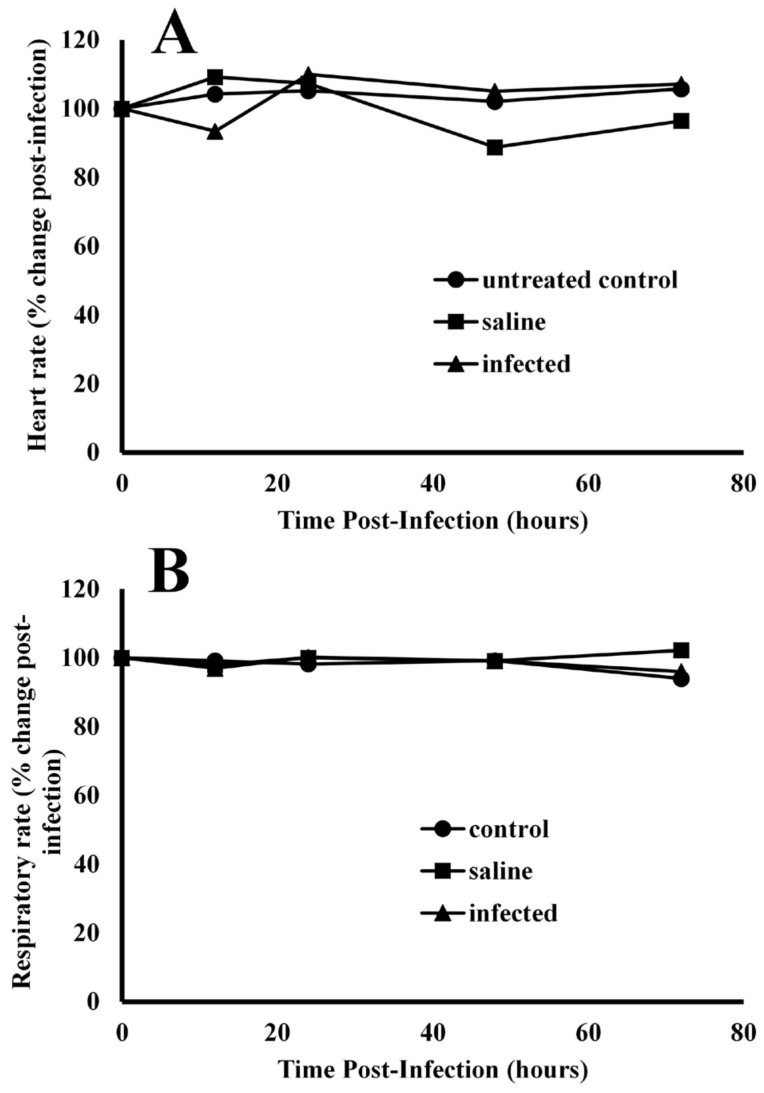
Comparison of cardiac and pulmonary function in alligators before and after infection with bacteria. After infection, the heart rate (**A**) and respiratory rate (**B**) were unchanged in alligators relative to baseline values before infection. The data are expressed as the means of four independent determinations.

**Effects of infection on heart rate and respiratory rates**—Experimental coelomic infection of alligators with bacteria exhibited no effects on heart rate (Figure 3A) or respiratory rate (Figure 3B). The baseline resting heart rate for alligators prior to infection was 50.3 ± 6.7 beats per minute. After infection of bacteria, the heart rates were 87.4 ± 12.8, 109.9 ± 8.5, 105.1 ± 15.6, and 107.1 ± 7.9% of baseline values at 12, 24, 48, and 72 h post-injection. Injection of alligators with pyrogen-free saline resulted in heart rates of 109.2 ± 10.7, 107.3 ± 9.6, 84.7 ± 9.1, and 96.4 ± 7.1 for the same time intervals. ANOVA test results showed that the population means for heart rate differed between time points for only one treatment, F(8, 36) = 2.276, *p* = 0.044. Further testing using a Bonferroni adjustment showed that the difference lay in the saline treatment group between 24 (M = 107.3, SD = 9.59) and 48 h (M = 88.8, SD = 10.3), *p* = 0.047.

The baseline respiratory rate for all alligators was 2.4 ± 0.1 breaths per minute. Injection with bacteria resulted in 97.0 ± 4.0, 100.2 ± 6.6, 99.1 ± 5.0, and 96.0 ± 3.2% of baseline respiratory rates at 12, 24, 48 and 72 h post-infection (Figure 3B). ANOVA test results showed no significant interaction effect of respiratory rate for each treatment–time combination, F(8, 36) = 1.165, *p* = 0.346.

**Figure 4:**
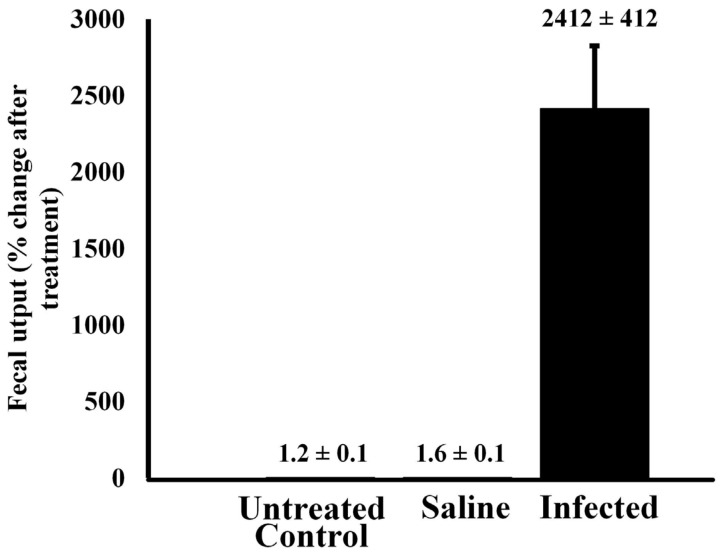
Fecal output of untreated alligators and animals injected with sterile saline or bacteria. Alligators were treated with either pyrogen-free saline or live bacteria, and their fecal output was measured 6 h after treatment and compared to that of untreated control animals. The data are expressed as the means ± standard deviations of four independent determinations.

**Effect of bacterial infection on fecal output**—Injection of alligators with bacteria resulted in a large increase in fecal output within 6 h of injection (Figure 4). A Welch’s ANOVA was conducted to compare results in fecal output between untreated control alligators and those treated with sterile saline or infected with 10^5^ CFU bacteria. The results indicated that there was a statistically significant change in fecal output from baseline between the three treatment groups, F(2, 4.044) = 14.1, *p* = 0.015. A Games–Howell post hoc test showed that there was no statistically significant difference in fecal output change between the control (M = 0.00614, SD = 0.007) and saline groups (M = 0.00246, SD = 0.0007), *p* = 0.605. However, the infected group showed significantly higher fecal output than the untreated control alligators (*p* = 0.022) and the saline-treated animals (*p* = 0.022).

Alligators injected with sterile saline exhibited no statistical change in fecal output relative to untreated control animals (*p* > 0.05). However, injection of 10^5^ CFU led to a 2100-fold increase in fecal output within 6 h relative to untreated controls (*p* < 0.00001).

## 4. Discussion

It has long been known that systemic infection of humans with Gram-negative bacteria produces symptoms of toxic shock [28,29,30] including high fever, increased oxygen consumption [31], increased heart rate [32], and elevated respiratory rate [33]. The biological effects are produced primarily due to the presence of lipopolysaccharide (LPS), an amphipathic molecule that comprises a significant portion of the outer membrane of Gram-negative bacteria [34]. This molecule is recognized by a variety of humoral and cellular components of the innate immune system [10]. Although alligators have well-defined innate immune mechanisms (reviewed in [35]), injection of juvenile alligators with 100 times the murine LD_50_ of LPS (based on body mass) produced no obvious biological effects. Alligators injected with LPS were not lethargic, exhibited no mortality, and showed no change in serum protein electrophoretic profiles relative to untreated animals (data not shown).

Acute systemic infections in mammals can be detected by examination of changes in expression of serum proteins. These changes are often observed and measured by clinical agarose serum protein electrophoresis [36], and are obvious in a broad spectrum of mammals including canines [37], pigs [38], and ruminants [39]. One of the major changes in serum protein profiles after infection is the increase in the α- and β-globulin groups of proteins. The α-globulin group contains inflammatory proteins such as α-lipoprotein, α1-antitrypsin, α1-acid glycoprotein, α2-macroglobin, and haptoglobin, while the β-globulins include transferrin, serum amyloid A, C-reactive protein, and fibrinogen [30]. Increases in the α- and β-globulin proteins are an indication of potential acute inflammation in birds [40] and mammals [41]. Another major change during infection and inflammation is a decrease in the amount of albumin in the serum, which may reflect selective loss of albumin due to renal or gastrointestinal changes or a decrease in hepatic synthesis [42]. Thus, a low ratio of albumin/globulin can be an indication of inflammation. The relatively high amounts of α- and β-globulins in uninfected alligators (Figure 1) may indicate a constant state of primed immunity. These results are supported by the alligator transcriptome results obtained from the livers of uninfected alligators and mice (Table 1, [43]). These results clearly show that a large fraction of the highest constitutively expressed genes in mice are related to metabolism. This reflects the importance of these genes to endotherms, as maintenance of body temperature is of the utmost importance [44]. In contrast, although metabolic genes are highly conserved in all vertebrates [21], many of the highest-expressed genes in the liver of unchallenged alligators are those related to innate immune defense (Table 1). Because alligators, like many ectotherms, have less sophisticated adaptive immune systems [18,19], they may express innate immune proteins constitutively at relatively high levels to prevent infection. The energetic trade-off of ectothermy allows more energy to be dedicated to constant expression of genes that code for the expression of proteins with innate immune function [45]. However, other reptiles do not seem to share this high level of constitutive immunological preparedness. For instance, the albumin/globulin of Russian tortoises (*Testudo horsfieldii*, n = 4) was shown to be 0.92–1.27 and for Burmese pythons (Python mollurus bivattus, n = 3) this ratio was 1.31–1.40 [46]. For three species of lizards in the genus *Gallotia*, the albumin/globulin ratios were 0.57–0.59 [47]. The albumin/globulin ratio for healthy green iguanas (*Iguana iguana*) was 0.76, and was 0.59 for green turtles (*Chelonia mydas*, [48]). This ratio decreased to 0.24 in iguanas that presented with clinical conditions of listlessness behavior, decreased appetite, muscle fasciculations, and evidence of bone abnormalities [49], which indicated that, in contrast to alligators, the serum protein profiles in the iguanas were capable of change (increased α- and β-globulins and decreased albumin) under conditions of clinical abnormalities [49]. For eight alligators examined in this study, the albumin/globulin ratio was 0.17 ± 0.04 (three shown, Figure 1A), which indicates higher expression of innate immune proteins under unchallenged immunological conditions. Interestingly, Coppo et al. [50] reported that albumin/globulin ratios for healthy broad-snouted caiman (*Caiman latirostris*, *n* = 109) and yacare caiman (*Caiman yacare*, *n* = 114) were 0.32 ± 0.07 and 0.30 ± 0.05, respectively, which indicates that other members of the Family Alligatoridae, and potentially other members of the Order Crocodylia, may exhibit the same high levels of immune proteins. This supports the idea that crocodylians have constitutively active innate immune systems [51,52]. In addition, these observations would explain the findings that both American alligators and broad-snouted caiman exhibit potent antibacterial properties [53,54] and high serum complement innate immune activities [14,55].

The idea that immunologically unchallenged alligators exhibit high constitutive levels of immune proteins was supported by results of experiments that demonstrated elevated expression of hepatic genes related to immunity (Table 1). In contrast, the livers of uninfected mice expressed more genes related to metabolism (Table 1). These differences highlight the energetic demands of endotherms to maintain body temperature as well as the need for crocodylians to sustain a high level of innate immunological vigilance. Furthermore, differential gene expression studies have shown that alligator hepatic gene expression does not undergo extensive acute alteration after bacterial infection [43].

We were surprised to find that metabolic rate in alligators decreased significantly after infection (Figure 2). In mammals and birds, metabolic rate increases substantially after acute infection due to the energetic demands of initiating an immunological assault on invading pathogenic microbes [56]. The increase in metabolism is activated to meet the energy required to recruit inflammatory cells to the site of infection [57], generate large quantities of reactive nitrogen and oxygen intermediates [58,59], and support proliferation of T-cell and B-cell populations [18,19,60]. However, it is expected that alligators would require the same energetic requirements to provide energy for an acute immune response.

To determine if the cause of the decreased metabolic rate after infection was due to a bradycardia effect, we measured heartbeat rates in alligators before and after infection Figure 3A). The resting heart rate of all alligators before treatment was 50.3 ± 6.6 bpm (range = 40–60 bpm), which is much higher than other reports of 26–43 bpm [61], 25–30 bpm [62], and 40 bpm [63] for juvenile American alligators. However, temperature is strongly associated with heart rate, and the alligators in the present study were maintained at 30 °C, while in all other studies alligators were maintained at ambient laboratory temperatures. We were again surprised to find that the decrease in metabolic rate was not associated with an decrease in heart rate. Instead, the heart rate remained constant for at least four days after infection (Figure 3A). In an attempt to determine if bradypnea was associated with the decreased oxygen consumption after infection, we measured respiratory rates in infected and uninfected animals (Figure 3B). Similarly, the respiratory rate at ambient temperature (30 °C) was 2.73 ± 0.51 breaths/min, which was similar to those measured by Coulson and Hernandez [64] at ambient temperature [64] (1.5–3.3 breaths/min, 23–25 °C) and Campos [65] (breaths/min, 32 °C). However, the respiratory rate also did not change after acute infection with bacteria (Figure 3B).

Joanen and McNeese [66] showed that healthy juvenile alligators process food and empty their digestive tracts every 24–48 h. In this study, we report that almost no fecal matter was expelled by untreated or saline-injected alligators within six hours after feeding (Figure 4). However, there was more than a 2000-fold increase in fecal output in animals experimentally infected with bacteria. Coulson and Hernandez [67] found that digestion requires considerable energy for processing and conversion. It is assumed that the infected alligators purge their digestive load to minimize the energy spent on digestion to redirect that resource to the immediate need for an immune response. This is not a general stress response as restraint stress and handling stress did not evoke the same response (data not shown). In addition, injection of animals with sterile saline did not increase fecal output (Figure 4).

Alligators, like other animals, must meet energetic demands for immune system activation and maintenance of an active state for a prolonged period of time until an immunological threat is neutralized. The energetic costs of immunity are high, and the energy used to invest immunity must be balanced against energy resources allocated for other biological processes [68,69,70]. The decrease in oxygen consumption observed without the commensurate reductions in heart and respiratory rates indicates that the infection did not simply depress all biological systems within the experimental animals. However, instead of increasing oxygen consumption during infection, alligators seem to redistribute energy from other biological functions to maintain or increase immune function. It has been suggested that the energy required for immune system function may limit biological processes (such as reproduction) in ectotherms [2]. During infection, alligators also appear to decrease activity of other biological functions, such as digestion, without reducing heart or respiratory rates, to temporarily use the energy to increase immunity to stop the immediate threat of infection. This energy can be used for the expansion of leukocyte populations in response to infection [71], activation of respiratory burst mechanisms to generate reactive oxygen species [72], chemotaxis of leucocytes and degranulation to release antimicrobial peptides at the site of infection [70], or any myriad of other immunological functions. This is a unique strategy of resource reallocation without any increase in metabolism that has not been described in any other animal.

Alligators also exhibit other interesting and primitive methods of defense that have not been described in other vertebrates. This was apparent during examination of one particular animal with a series of deep, parallel wounds on the dorsum that were obviously inflicted by a boat propeller. Although the wounds had completely healed, it was observed that they were heavily impacted with marsh mud and sediment and that the alligator had deposited many layers of fibrin, approximately 2–2.5 cm thick, that isolated the wound from the surrounding tissues (Dr. Val Lance, unpublished observation). This process was later described by Huchzermeyer and Cooper [73] as fibriscess, as they described deposition of fibrin around areas of localized chronic infection in crocodiles, presumably to exclude the infected area from adjacent tissues and avoid systemic septicemia. This method of host defense is reminiscent of the melanization response by arthropods, in which layers of insoluble melanin are produced to insulate infectious agents from healthy tissue [74]. These methods require relatively low amounts of energy input and effectively isolate the threat for an indefinite time frame. The data presented in this study reveal insights into the other unique responses of alligators to systemic infection.

## 5. Conclusions

Because alligators are ectothermic animals, they lack the energetic demands of maintaining body temperature by metabolic means. This allows these animals to spend more effort to maintain high levels of immunological genes. This is important because alligators exhibit conspecific aggressive behavior in the defense of territories, defense of eggs and hatchlings, etc., [52] and these behaviors can result in serious injuries that require immediate immunological attention. The importance of immunological preparedness is highlighted by the fact that alligators live in aquatic environments that harbor a multitude of potentially infectious microbial organisms that could easily colonize compromised and wounded tissues. In addition, the data presented in this study indicate that alligators have also developed a strategy of minimizing the functions of other organ systems, such as the digestive tract, to focus energy on eliminating infections. To our knowledge, this is the first report of a directed effort to reduce oxygen consumption during an infection. However, this strategy might only be enacted when the animals are constrained to constant temperature, because alligators are known to have the ability to induce a behavioral febrile response during infection [16].

## Figures and Tables

**Table 1:** Constitutive hepatic gene expression in mice (*Mus muluscus*) and American alligators (*Alligator mississippiensis*).

Transcriptome	Mouse	Alligator
Top highest-expressed genes analyzed	100	100
# metabolic process genes in highest 100 expressed	55	19
# immunity genes in highest 100 expressed	11	29
Total transcripts	7,262,841	4,449,289
Transcripts for metabolic processes	4,153,760 (57.2%)	870,276 (19.6%)
Transcripts for immunity	796,288 (11.0%)	1,494,348 (33.6%)

## Data Availability

The data presented in this study are available on request from the corresponding author.
